# Perceptions of Parenting Challenges and Career Progression Among Physician Faculty at an Academic Hospital

**DOI:** 10.1001/jamanetworkopen.2020.29076

**Published:** 2020-12-10

**Authors:** Helen Kang Morgan, Kanakadurga Singer, James T. Fitzgerald, Kirk J. Brower, Brooke M. Spencley, Lauren E. Owens, Heather L. Burrows

**Affiliations:** 1 Department of Obstetrics and Gynecology, University of Michigan, Ann Arbor; 2 Department of Learning Health Sciences, University of Michigan, Ann Arbor; 3 Department of Pediatrics, University of Michigan, Ann Arbor; 4 Department of Psychiatry, University of Michigan, Ann Arbor; 5 Department of Surgery, University of Wisconsin, Madison

## Abstract

This survey study compares the perceptions of male and female physician faculty members who have children regarding parenting challenges and career progression at an academic hospital.

## Introduction

Achieving gender parity in medicine requires identification of the barriers contributing to unequal career progression for women. Individual factors such as increased childcare responsibilities are already well documented^1^; it is now time to examine systems barriers such as institutional culture. The goals of this study were, first, to ascertain physician faculty’s perceptions of parenting challenges and career progression and, second, to identify differences in perceptions between male and female faculty members.

## Methods

A 31-item Likert scale survey (eMethods 1 and 2 in the Supplement) was developed by adapting questions from previous surveys pertaining to pregnancy, parenting,^2^ and perceptions of how parenting affects employment promotions.^3^ Responses were classified as complete, partial, or eligible “noninterview” per the American Association for Public Opinion Research (AAPOR) reporting guideline. Additional demographic questions included respondent sex, department, and parental status. Participants who responded affirmatively to the parent question were asked to describe how parenting commitments have affected their participation in service, scholarship, and leadership opportunities. The Michigan Medicine institutional review board exempted this study from the need for approval because the responses were anonymous; respondents did not provide informed consent.

The survey was electronically administered to all physician faculty at Michigan Medicine, University of Michigan Medical School, Ann Arbor, in October 2019, and 4 weekly email reminders for responses were sent. Mean comparisons between male and female respondents were analyzed using 2-tailed *t* tests and analyses of variance; post hoc analyses used the Tukey-Kramer honestly significant difference test. The JMP Pro, version 14.2.0 software package (SAS Institute Inc) was used for statistical analysis. We used a Bonferroni correction to set the 2-sided *P* value at .007 to indicate a significant difference.

## Results

The overall response rate to the survey was 52.4% (1085 of 2069 respondents). Of the 1085 respondents, 953 (87.8%) identified as being parents, 632 (58.2%) as female, and the majority (682 [62.9%]) completed postgraduate training after 2005 (Table). Among respondents, 992 (91.4%) indicated their specialty (Table).

**Table.** Respondent Demographic Characteristics

Characteristic	No. (%)		
	Male (n = 453)	Female (n = 632)	Total (N = 1085)
Parent	416 (43.7)	537 (56.3)	953 (87.8)
Nonparent	37 (28.0)	95 (72.0)	132 (12.2)
Year of completion of postgraduate training^a^			
Before 1991	42 (59.2)	29 (40.8)	71 (7.3)
1991-1995	38 (63.3)	22 (36.7)	60 (6.2)
1996-2000	53 (51.0)	51 (49.0)	104 (10.7)
2001-2005	47 (37.6)	78 (62.4)	125 (12.9)
2006-2010	74 (42.3)	101 (57.7)	175 (18.0)
2011-2015	80 (34.6)	151 (65.4)	231 (23.8)
After 2015	78 (38.0)	127 (62.0)	205 (21.1)
Specialty^b^			
Anesthesiology	33 (46.5)	38 (53.5)	71 (7.2)
Dermatology	2 (16.7)	10 (83.3)	12 (1.2)
Emergency medicine	22 (44.9)	27 (55.1)	49 (4.9)
Family medicine	14 (29.2)	34 (70.8)	48 (4.8)
General surgery	35 (70.0)	15 (30.0)	50 (5.0)
Internal medicine	122 (44.5)	152 (55.5)	274 (27.6)
Neurology	26 (70.3)	11 (29.7)	37 (3.7)
Neurosurgery	3 (60.0)	2 (40.0)	5 (0.5)
Obstetrics and gynecology	9 (17.6)	42 (82.4)	51 (5.1)
Ophthalmology	20 (41.7)	28 (58.3)	48 (4.8)
Otolaryngology	14 (63.6)	8 (36.4)	22 (2.2)
Pathology	11 (47.8)	12 (52.2)	23 (2.3)
Pediatrics	31 (22.5)	107 (77.5)	138 (13.9)
Physical medicine and rehabilitation	12 (41.4)	17 (58.6)	29 (2.9)
Psychiatry	15 (35.7)	27 (64.3)	42 (4.2)
Radiation oncology	6 (46.2)	7 (53.8)	13 (1.3)
Radiology	20 (50.0)	20 (50.0)	40 (4.0)
Surgical specialties	11 (84.6)	2 (15.4)	13 (1.3)
Urology	8 (47.1)	9 (52.9)	17 (1.7)

^a^ Of the total respondents, 89.5% (971 of 1085) indicated the year of completion of postgraduate training.

^b^ Of the total respondents, 91.4% (992 of 1085) indicated their specialty.

The Figure shows responses pertaining to pregnancy, parenting, promotion, and communication with leadership analyzed by sex. Both male and female respondents reported a positive culture pertaining to pregnancy, with department leadership supportive of faculty and schedule flexibility related to pregnancy. Respondents reported that parenting negatively affects promotion for women but not for men. Neither male nor female respondents reported that they felt comfortable discussing work-life integration with their divisional or departmental leadership. Male respondents responded more affirmatively to the statements that male physicians who are parents fall behind in the promotion process and that they are more comfortable discussing work-life integration with leadership.

**Figure.**
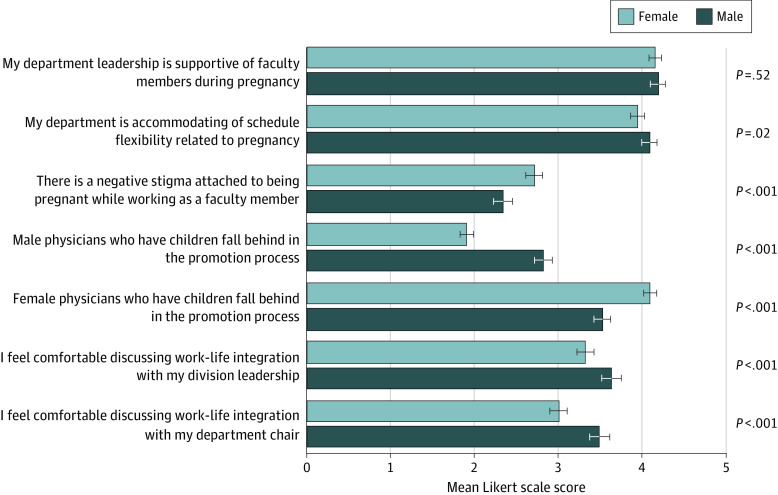
Pregnancy, Promotion, and Support Mean ratings on 5-point Likert scale for survey questions pertaining to pregnancy, parenting, promotion, and communication with leadership, by sex. Whiskers indicate SD. A Likert scale score of 1 indicates strongly disagree; 2, disagree; 3, neither agree nor disagree; 4, agree; and 5, strongly agree.

Among 953 physician parents, women were more likely than men to respond that, because of specific parenting commitments, they “have turned down a project” (women, 285 [53.1%]; men, 181 [43.5%]; *P* = .004) or had “not participated in an institutional or departmental committee” (women, 256 [47.7%]; men, 155 [37.2%]; *P* = .002). Women were more likely than men to report that they had “turned down a leadership role,” but the difference was not statistically significant (women, 129 [24.0%]; men, 78 [18.8%]; *P* = .06). Both male and female respondents answered the following statement at similarly high rates: “I have not presented at a national conference” (women, 303 [56.4%]; men, 215 [1.7%]; *P* = .15).

## Discussion

Our survey study of physician faculty identified work-family tensions and challenges related to communication. It is notable that all respondents indicated that they were not comfortable discussing work-life integration issues with their leadership and that women were less likely to be comfortable in such circumstances than men. A limitation of this work is that we did not specifically ask why physician faculty did not feel comfortable discussing these challenges with their leadership. Nevertheless, effective approaches for gender parity should include engaged leadership. The work of all physicians is temporally relevant given that the coronavirus disease 2019 pandemic has disproportionately affected parents, particularly women.^4^ The disruptions introduced by the pandemic present an opportunity for those in leadership to play a role in improving institutional culture by acknowledging and supporting parenting challenges.

The survey results were also striking in noting how frequently physician parents turned down scholarship and leadership opportunities even before the pandemic. Work-home conflicts are a strong contributor to physician burnout.^5^ Now is a propitious time to evaluate how recent changes, such as virtual conferences and meetings, can be maintained to better support physicians juggling family responsibilities. These unprecedented times provide an opportunity for academic institutions to address systems barriers for parents and especially for women.

## Notes

Supplement. **eMethods 1.** Survey **eMethods 2.** Survey Development **eReferences.**
