# Error in Results Section

**DOI:** 10.1001/jamanetworkopen.2020.36665

**Published:** 2021-01-15

In the Research Letter titled “Perceptions of Parenting Challenges and Career Progression Among Physician Faculty at an Academic Hospital,”^1^ published December 10, 2020, a percentage in the Results section was incorrectly reported. In the last sentence of that section, the percentage for men should have appeared as 51.7% instead of 1.7%. This article has been corrected.^1^

## References

[1] Morgan HK, Singer K, Fitzgerald JT, Perceptions of parenting challenges and career progression among physician faculty at an academic hospital. JAMA Netw Open. 2020;3(12):e2029076. doi:10.1001/jamanetworkopen.2020.29076 33301013 PMC7729426

