# Supplementary material for: Perceptions of Parenting Challenges and Career Progression Among Physician Faculty at an Academic Hospital
Source: JAMA Netw Open. 2020 Dec 10;3(12):e2029076. doi: 10.1001/jamanetworkopen.2020.29076 (PMC7729426; doi:10.1001/jamanetworkopen.2020.29076)
Supplement: Supplement. — eMethods 1. Survey eMethods 2. Survey Development eReferences. [file jamanetwopen-e2029076-s001.pdf]

Supplemental Online Content

Morgan HK, Singer K, Fitzgerald JT, et al. Perceptions of parenting challenges and career progression among physician faculty at an academic hospital. *JAMA Netw Open.* 2020;3(12):e2029076. doi:10.1001/jamanetworkopen.2020.29076

eMethods 1. Survey

eMethods 2. Survey Development

eReferences.

This supplemental material has been provided by the authors to give readers additional information about their work.

© 2020 Morgan HK et al. *JAMA Network Open.*

eMethods 1. Survey

1) Are you a parent
    a. Yes
    b. No
2) Please indicate the age of your child or children (check all that apply)
    a. <6
    b. 6-18
    c. >18
3) Have you ever been pregnant?
    a. Yes
    b. No
4) Have you been pregnant while working as a faculty member at the University of Michigan?
    a. Yes
    b. No
5) Who is your primary childcare during work hours? (or was your primary child care if your child(ren) are older) - Selected Choice
    a. Individual hired help (nanny, babysitter, etc)
    b. Daycare or other group facility
    c. Spouse
    d. Other family member
    e. None
    f. Other (free text)
6) Who is your primary childcare during work hours? (or was your primary child care if your child(ren) are older) - Other: - Text
7) Are you or your partner planning a pregnancy in the next 5 years?
    a. Yes
    b. No

---

8) What resources would be helpful to you as you are thinking about pregnancy? - Selected Choice
    a. Departmental/University Policies on Scheduling Personal Medical Appointments
    b. Assistance with selection of Obstetrical and/or CNM provider
    c. University of Michigan policy on parental leave
    d. University of Michigan Health Plan Fertility Treatment Coverage
    e. General Information Regarding Fertility Treatment/Preservation
    f. Other:
9) What resources would be helpful to you as you are thinking about pregnancy? - Other: - Text

---

10) Do you feel adequately knowledgeable regarding the University of Michigan policy on parental leave (including maternal, paternal, and adoption)?
    a. Definitely yes
    b. Probably yes
    c. Might or might not
    d. Probably not
    e. Definitely not
11) Do you feel adequately knowledgeable regarding your departmental policy and practices on parental leave (including maternal, paternal or adoption?)
    a. Definitely yes
    b. Probably yes
    c. Might or might not
    d. Probably not
    e. Definitely not

© 2020 Morgan HK et al. *JAMA Network Open.*

12) Do you feel adequately knowledgeable regarding Michigan Medicine resources for lactation support?
    a. Probably yes
    b. Might or might not
    c. Probably not
    d. Definitely not
13) My department leadership is supportive of faculty members during pregnancy
    a. Strongly agree
    b. Somewhat agree
    c. Neither agree nor disagree
    d. Somewhat disagree
    e. Strongly disagree
14) My department is accommodating of schedule flexibility related to pregnancy
    a. Strongly agree
    b. Somewhat agree
    c. Neither agree nor disagree
    d. Somewhat disagree
    e. Strongly disagree
15) There is a negative stigma attached to being pregnant while working as a faculty member
    a. Strongly agree
    b. Somewhat agree
    c. Neither agree nor disagree
    d. Somewhat disagree
    e. Strongly disagree
16) Male physicians who have children fall behind in the promotions process
    a. Strongly agree
    b. Somewhat agree
    c. Neither agree nor disagree
    d. Somewhat disagree
    e. Strongly disagree
17) Female physicians who have children fall behind in the promotions process
    a. Strongly agree
    b. Somewhat agree
    c. Neither agree nor disagree
    d. Somewhat disagree
    e. Strongly disagree
18) I feel comfortable discussing work-parenting-integration issues with my division leadership
    a. Strongly agree
    b. Somewhat agree
    c. Neither agree nor disagree
    d. Somewhat disagree
    e. Strongly disagree
19) I feel comfortable discussing work-parenting-integration issues with my department chair
    a. Strongly agree
    b. Somewhat agree
    c. Neither agree nor disagree
    d. Somewhat disagree
    e. Strongly disagree

© 2020 Morgan HK et al. *JAMA Network Open.*

20) Because of parenting or parenting commitments, I have: (select all that apply) - Selected Choice
    a. Turned down a project at my institution
    b. Turned down a leadership role at my institution
    c. Turned down a position at my institution
    d. Not participated in an institutional or departmental committee
    e. Not presented at a national meeting
    f. Felt compelled to take on a project
    g. Felt compelled to take on a leadership role at my institution
    h. Felt more efficient during my work day
    i. Other
    j. None of the above
21) Because of parenting or parenting commitments, I have: (select all that apply) - Other – Text
22) Is there anything you wish to share about your experience of pregnancy or parenting as a physician? (free text)
23) I wish I had more guidance on local resources about the following categories before and/or during pregnancy: (check all that apply) - Selected Choice
    a. Flexible Work Schedules
    b. Lactation Facilities
    c. Pet Care
    d. Meal Planning
    e. Reproductive, Endocrine and Infertility Services
    f. Unpredictable or emergency childcare options (eg. illness or snow days)
    g. Childcare
    h. Parental Leave
    i. Grocery Delivery
    j. Kid Friendly Activities in Ann Arbor
    k. Cleaning Services
    l. Dry Cleaning
    m. Other
24) I wish I had more guidance on local resources about the following categories before and/or during pregnancy: (check all that apply) - Other – Text
25) How early would you recommend establishing childcare plans (i.e. enroll in day care, hire a nanny, etc.)?
    a. Before pregnancy
    b. First trimester
    c. Second trimester
    d. Third trimester
26) What local services, day cares, or childcare options would you recommend to future physician parents? (free text)

---

27) What year did you complete your postgraduate training? (free text)
28) What year did you begin working at the University of Michigan as a faculty member? (free text)

29) What department do you currently work in?
  a. Obstetrics & Gynecology
  b. Anesthesiology
  c. Family Medicine
  d. Pediatrics – General
  e. Pediatrics - Hospital Medicine
  f. Pediatrics - Pulmonology
  g. Pediatrics - Neonatal-Perinatal Medicine
  h. Pediatrics - Developmental Behavioral
  i. Pediatrics – Neurology
  j. Pediatrics – Cardiology
  k. Pediatrics - Gastroenterology
  l. Pediatrics - Adolescent Medicine
  m. Pediatrics - Hematology/Oncology
  n. Pediatrics - Infectious Diseases
  o. Pediatrics - Critical Care Medicine
  p. Pediatrics - Genetics, Metabolism & Genomic Medicine
  q. Internal Medicine - Hospital Medicine
  r. Internal Medicine - General Medicine
  s. Internal Medicine – Rheumatology
  t. Internal Medicine - Infectious Diseases
  u. Internal Medicine - Hematology and Oncology
  v. Internal Medicine - Pulmonary and Critical Care
  w. Internal Medicine – Nephrology
  x. Internal Medicine - Gastroenterology and Hepatology
  y. Internal Medicine - Metabolism, Endocrinology & Diabetes
  z. Internal Medicine - Geriatric and Palliative Medicine
  aa. Internal Medicine - Cardiovascular Medicine
  bb. Internal Medicine - Allergy and Clinical Immunology
  cc. Orthopaedic Surgery
  dd. Physical Medicine and Rehabilitation
  ee. Neurology
  ff. General Surgery
  gg. Surgery
  hh. Psychiatry
  ii. Emergency Medicine
  jj. Radiation Oncology
  kk. Radiology
  ll. Pathology
  mm. Ophthalmology & Visual Sciences
  nn. Cardiac Surgery
  oo. Urology
  pp. Otolaryngology - Head and Neck Surgery
  qq. Neurosurgery
  rr. Dermatology
  ss. Vascular Surgery
  tt. Thoracic Surgery
30) What is your gender identity? - Selected Choice
  a. Male
  b. Female
31) What is your gender identity? - Other - Text

© 2020 Morgan HK et al. *JAMA Network Open.*

eMethods 2. Survey development

For survey design, the authors adapted questions from previously published surveys pertaining to parental leave,[1] departmental support for pregnancy and parenting,[2] and perceptions of how parenting affect promotion processes,[3] contributing to content validity. Of note, the results pertaining to parental leave were not included in this manuscript. In order to optimize response process validity, multiple rounds of piloting the survey were performed with physician faculty and content experts, leading to revisions.

The survey distribution was a list-based sample of specifically named persons. An email list of physician faculty was generated in October 2019 for this study by the Michigan Medicine Department of Faculty Affairs. This email list was created by querying all instructional and clinical track faculty with MD and DO degrees. The first email invitation to participate was sent by the Executive Vice Dean of Academic Affairs, with follow-up weekly email reminders sent by the study authors. The email invitations contained an anonymous survey link. Responses were classified as complete, partial, or eligible "non-interview" per the American Association of Public Opinion Research reporting guidelines.[4] Partial responses were included in these results if questions pertaining to gender, department, and departmental support for pregnancy and parenting were completed.

The JMP® Pro 14.2.0, SAS Institute Inc. statistical package was used for survey analysis. A priori levels of significance were not established prior to the survey distribution, and a Bonferroni correction was used to set the p-value at 0.007 (0.05/7).

eReferences

1. Levinson W, Kaufman K, Bickel J. Part-time faculty in academic medicine: present status and future challenges. *Ann Intern Med.* 1993;119(3):220-225.
2. Mundschenk MB, Krauss EM, Poppler LH, et al. Resident perceptions on pregnancy during training: 2008 to 2015. *Am J Surg.* 2016;212(4):649-659.
3. Rangel EL, Lyu H, Haider AH, Castillo-Angeles M, Doherty GM, Smink DS. Factors Associated With Residency and Career Dissatisfaction in Childbearing Surgical Residents. *JAMA Surg.* 2018;153(11):1004-1011.
4. American Association for Public Opinion Research. Standard Definitions: Final Dispositions of Case Codes and Outcome Rates for Surveys. [Internet]. 2016; https://www.aapor.org/AAPOR_Main/media/publications/Standard-Definitions20169theditionfinal.pdf. Accessed October 2, 2020.

© 2020 Morgan HK et al. *JAMA Network Open.*
